# IgE responses to multiple allergen components among school-aged children in a general population birth cohort in Tokyo

**DOI:** 10.1016/j.waojou.2020.100105

**Published:** 2020-02-25

**Authors:** Kiwako Yamamoto-Hanada, Magnus P. Borres, Magnus K. Åberg, Limin Yang, Tatsuki Fukuie, Masami Narita, Hirohisa Saito, Yukihiro Ohya

**Affiliations:** a Allergy Center, National Center for Child Health and Development, Tokyo, Japan; b Thermo Fisher Scientific, Uppsala, Sweden; c Department of Women's and Children's Health, Uppsala University, Uppsala, Sweden; d Department of Allergy and Immunology, National Research Institute for Child Health and Development, Tokyo, Japan

**Keywords:** Allergy, Asthma, Atopic dermatitis, Child, Cohort, Eczema, IgE, ISAAC, ISAC, Prospective birth cohort, Rhinitis, Sensitization, Wheeze, CCD, Cross-reactive carbohydrate determinant, CRD, Component-resolved diagnostics, HDM, House dust mite, IgE, Immunoglobulin E, ISAAC, The International Study of Asthma and Allergies in Childhood, ISAC, Immuno-solid-phase Allergen Chip, JECS, The Japan Environment and Children's Study, PC, Principal component, PCA, Principal component analysis, PR, Pathogenesis-related, sIgE, Allergen-specific IgE, UK, The United Kingdom, US, The United States

## Abstract

**Background:**

Response patterns to allergen components among Japanese children have not been studied extensively.

**Objective:**

Our aim was to examine the differences in sensitization patterns at ages 5 years and 9 years to identify longitudinal changes in the degree and patterns of sensitization in a birth cohort of Japanese children.

**Methods:**

Our study enrolled 984 children at aged 5 years between 2008 and 2010, and 729 children aged 9 years between 2012 and 2014. Allergic diseases were assessed using the ISAAC and UK Working Party's Diagnostic Criteria. Serum-specific IgE titers to allergen components were measured by multiplex array ImmunoCAP ISAC when the children were aged 5 and 9 years. Principal component analysis (PCA) was performed to characterize IgE sensitization to allergen components.

**Results:**

The prevalence of allergic rhinitis increased considerably over time (10.6%–31.2%). Furthermore, the sensitization prevalence to allergen-specific IgE (sIgE) also increased from 57.8% at age 5 years to 74.8% at age 9 years. IgE sensitization prevalence to Der f 1 (mites) was 42.1% at age 5 years and 54.3% at age 9 years. Furthermore, children were highly sensitized to Cry j 1 (Japanese cedar) (32.8% at age 5 years and 57.8% at age 9 years). Principle component analysis showed that sensitization to PR-10 cross-reactive components was independent of sensitization to mite and that no children acquired sensitization to pollen before acquiring sensitization to mite.

**Conclusions:**

The prevalence of allergic rhinitis and related allergen components increased from age 5 years to age 9 years in Japanese children.

## Introduction

Allergic diseases have been increasing worldwide.^1^ Prospective longitudinal cohort studies examining the trajectory of allergic disease prevalence are rare in Japan; however, a cross-sectional study found that 10.5–18.2% of 6 to 14-year-old children in Tokyo had asthma symptoms.^2^ Although our previous study characterized the phenotype of wheezing/asthma and eczema/atopic dermatitis in Japanese children,^3^, ^8^ the course of rhinitis and sensitization among Japanese children has not been studied prospectively.

Allergen-specific immunoglobulin E (IgE) is integral to the pathogenesis of allergic disorders. An allergic reaction to an allergen is preceded by IgE sensitization to the allergen. The detection of allergens before sensitization is important in evaluating allergic diseases in patients with a clinical history of allergy for diagnostic purposes.^4^ Multiplex assays designed to detect IgE antibodies to multiple allergen components in human sera have been introduced into epidemiological studies,^5^, ^6^ and they have enabled the evaluation of sensitization profiles to over 100 allergen components in individuals. In Japan, 73.9% of pregnant women were sensitized to some allergen, and about half had an IgE titer indicating positivity to Japanese cedar pollen (55.6%) and mite component Der p 1 (48%).^7^ Sensitization patterns to allergen components vary from before elementary-school age, but no studies on this topic have thus far been published in Japan.

Variations in climate, geography, seasons, urbanization, and time periods affect the quantity of allergens, the prevalence of allergic diseases, and sIgE-sensitization.^9^ The aim of this study was to examine the changes in allergen-sensitization patterns from age 5 years–9 years in children living in Tokyo by using a multiplex component array.

## Methods

### Study design, setting and participants

This prospective birth cohort study of the general population, called the Tokyo Children's Health, Illness and Development study study (T-Child study),^3^, ^8^, ^10^, ^11^ was conducted at the National Center for Child Health and Development (NCCHD). Participants will be followed-up until they reach adulthood. The inclusion criteria were pregnant women who were followed at the NCCHD during pregnancy. In total, 1701 pregnant women were recruited at the National Center for Child Health and Development between 2003 and 2005, and 1550 newborns were included in the cohort. Children from multiple pregnancies were excluded. In total, 984 children aged 5 years between 2008 and 2010, and 729 children aged 9 years between 2012 and 2014 were examined. The final cohort consisted of 651 children with ISAC measurements for both time points.

The T-CHild Study was conducted in compliance with the Ethical Guidelines for Epidemiological Research proposed by the Ministry of Health, Labour and Welfare of Japan.

### Questionnaire and physical examination

A questionnaire was administered to parents to assess their child's exposure and health outcomes at ages 5 years and 9 years. Data on the outcomes for asthma, wheeze, eczema, and rhinitis were obtained from the International Study of Asthma and Allergies in Childhood (ISAAC) questionnaire.^12^ The Japanese translation of ISAAC used in this study was validated in accordance with the ISAAC protocol with the help of professors Ross Anderson and Hywel Williams, who are members of the ISAAC steering committee. The diagnosis of eczema was assessed by a physician using the U.K. Working Party's Diagnostic Criteria.^13^, ^14^, ^15^

### Blood sampling and IgE component analysis

Venous blood samples were obtained from children aged 5 years and 9 years. Serum-specific IgE titers to allergen components were analyzed using ImmunoCAP ISAC, a multiplex array (Phadia AB, Uppsala, Sweden).^16^, ^17^ Allergen components in the ImmunoCAP ISAC kit were identified by Thermo Fisher Scientific and are commercially available worldwide. ImmunoCAP ISAC enables measurement of IgE titers using a fixed panel of the 112 most relevant allergen components from 51 sources in a single test. The fixed panel for children aged 9 years was updated from that used for children aged 5 years, and 90 components were available on both panels. The IgE antibody levels were measured within a range of 0.3–100 ISU-E (ISAC Standardized Units). A specific IgE value ≥ 0.3 ISU was considered as positive. ImmunoCAP ISAC is a semi-quantitative test with three categories for specific IgE values (0.3 < ISU<1, 1 < ISU<15, and 15ISU). Subjects positive to any of the allergens on the ImmunoCAP ISAC were considered as having IgE sensitization. All measurements were done by a contract laboratory company (Thermo Fisher Scientific).

### Statistical analysis

First, a descriptive analysis of the prevalence of allergic features was performed. All subsequent analyses and Fig. 2, Fig. 3, Fig. 4, Fig. 5, Fig. 6 were based on data from the 651 subjects with ISAC measurements for both time points.

**Fig. 1:**
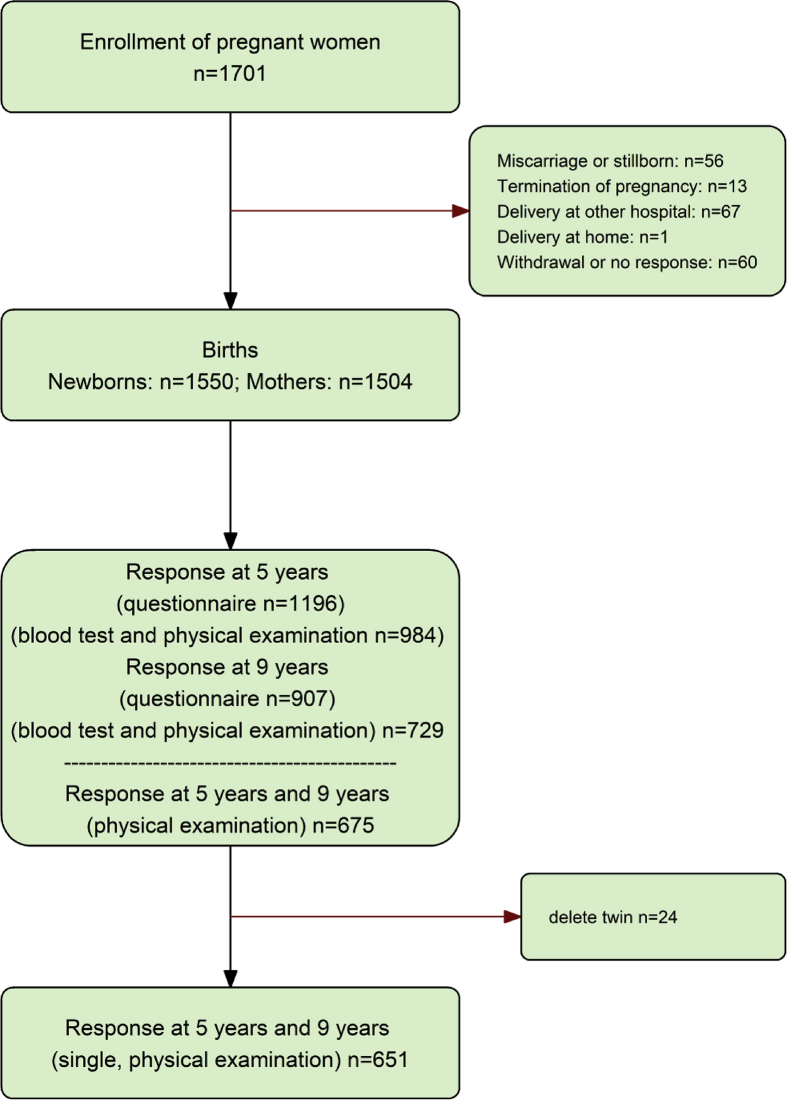
Study flow chart. Of the 1550 neonates enrolled in the T-Child study, 984 and 729, children aged 5 years and 9 years, respectively, were surveyed with a questionnaire and submitted a blood sample for analysis; 651 children had ISAC results for both time points

**Fig. 2:**
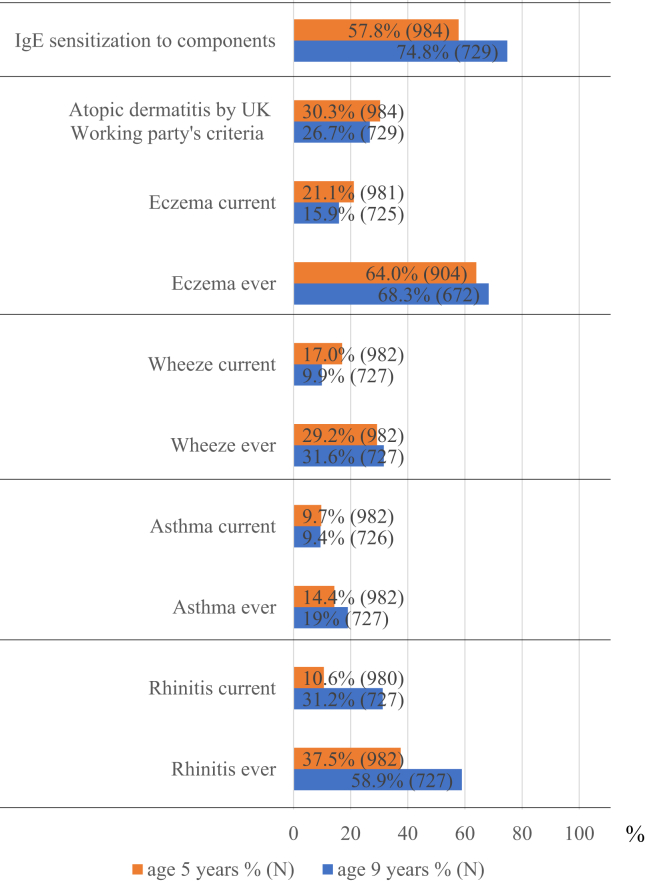
Prevalence of allergic features at ages 5 and 9 years. The prevalence of current allergic rhinitis increased considerably from 10.6% at age 5 years to 31.2% at age 9 years. On the other hand, the prevalence of current asthma was relatively stable at the two time points (9.7%–9.4%), and the prevalence of current wheeze decreased (17.0%–9.9%). Furthermore, the prevalence of sIgE sensitization also increased from 57.8% at age 5 years to 74.8% at age 9 years. On the other hand, the prevalence of current eczema and physician's diagnosis of eczema decreased from 21.1% to 30.3% at age 5 years to 15.9% and to 26.7% at age 9 years, respectively

**Fig. 3:**
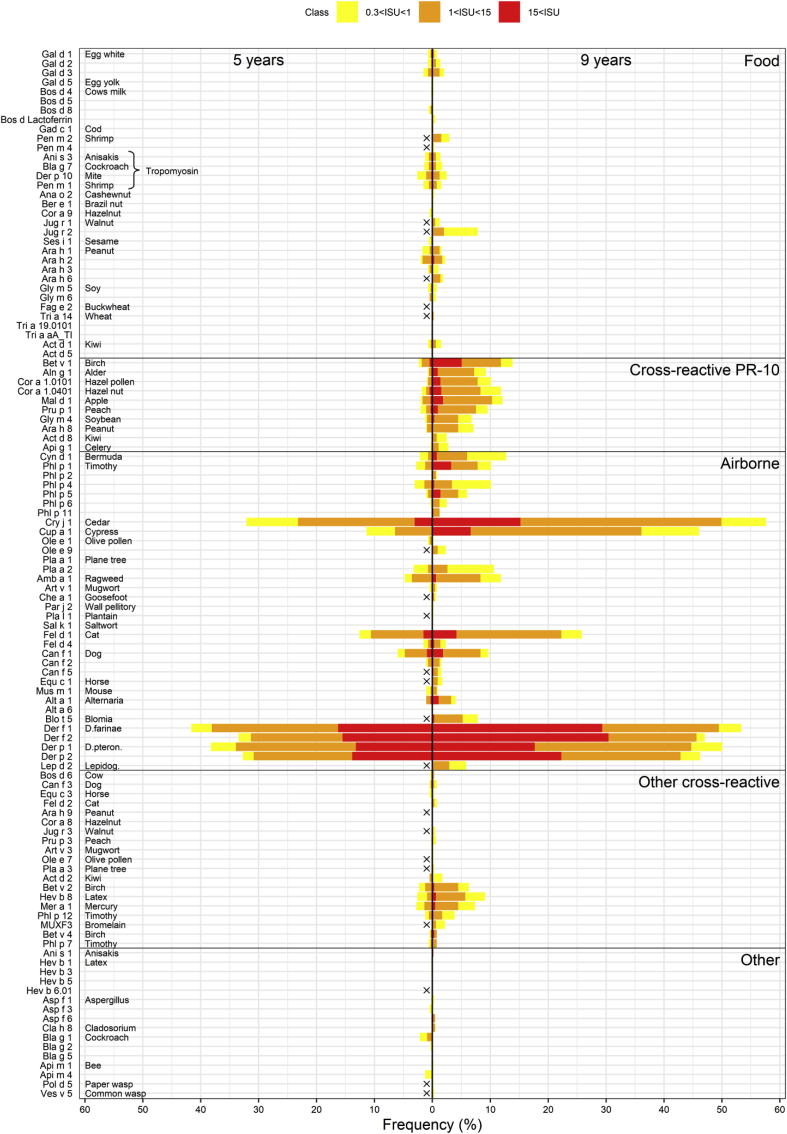
Prevalence of IgE sensitization to ISAC components in subjects aged 5 and 9 years. Components not presented on the chip used for measurements at 5 years are marked by a black "x." Gal d 1 sensitization to food allergens was found in 0.8% subjects at age 9 years. Jug r 1, 2, and 3-sensitization was 1.2%, 8.0%, and 0.4%, respectively, in subjects at this age. IgE sensitization to Ara h 2 (peanut) and Tri a 19.0101 (ω-5 gliadin, wheat) was found in 2.2% and 0% of the subjects, respectively, at age 9 years. Bal g 7 (cockroach, a tropomyosin) sensitization was found in 1.1% of subjects at age 5 years and 2.1% at age 9 years. With respect to PR-10 protein-related allergens, the proportion of IgE sensitization to Bet v 1 (birch) was 2.2% at age 5 years and 13.9% at age 9 years. In subjects aged 9 years, increased sensitization to Mal d 1 (apple), Pru p 1 (peach), and Gly m 4 (soy) was also evident. With respect to airborne allergens, the subjects were also highly sensitized to Cry j 1 (Japanese cedar) and Cup a 1 (Arizona cypress) (32.8% and 12.3% at age 5, 57.8% and 46.1% at age 9, respectively). The prevalence of IgE sensitization to Der f 1 (mite) at ages 5 and 9 years was 42.1% and 54.3%, respectively. Fel d 1 and Can f 1 sensitization occurred in 26.2% and 9.2% of subjects at age 9 years, respectively. Sensitization to another animal allergen, Mus m 1, occurred in 0.8% of the subjects at age 5 years and 1.0% of the subjects at age 9 years

**Fig. 4:**
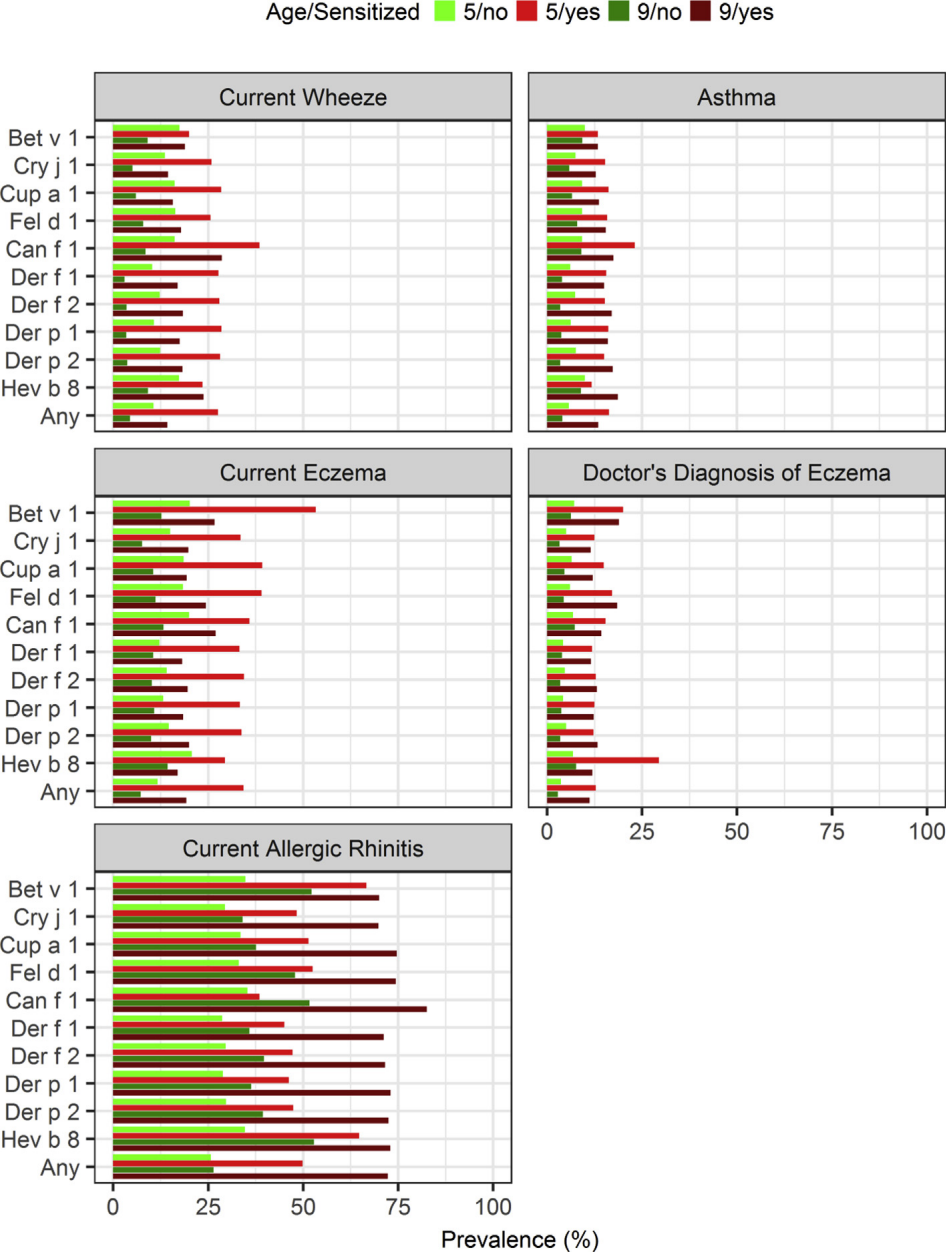
Prevalence of allergic diseases by age and sensitization. "Any" indicates sensitization to any of the other allergen components in the figure. Among these, the prevalence of current wheeze, eczema, and asthma decreased between the ages of 5 and 9 years. Among sensitized subjects, wheeze and current eczema also decreased, but less markedly. Bet v 1-sensitized subjects showed a higher prevalence of current eczema than the Cri j 1-sensitized subjects. Rhinitis increased with age in both groups although the increase was greater in the sensitized group. Approximately 75% of subjects at age 9 year who had been sensitized to Cry j 1 or Der f 1 had rhinitis

**Fig. 5:**
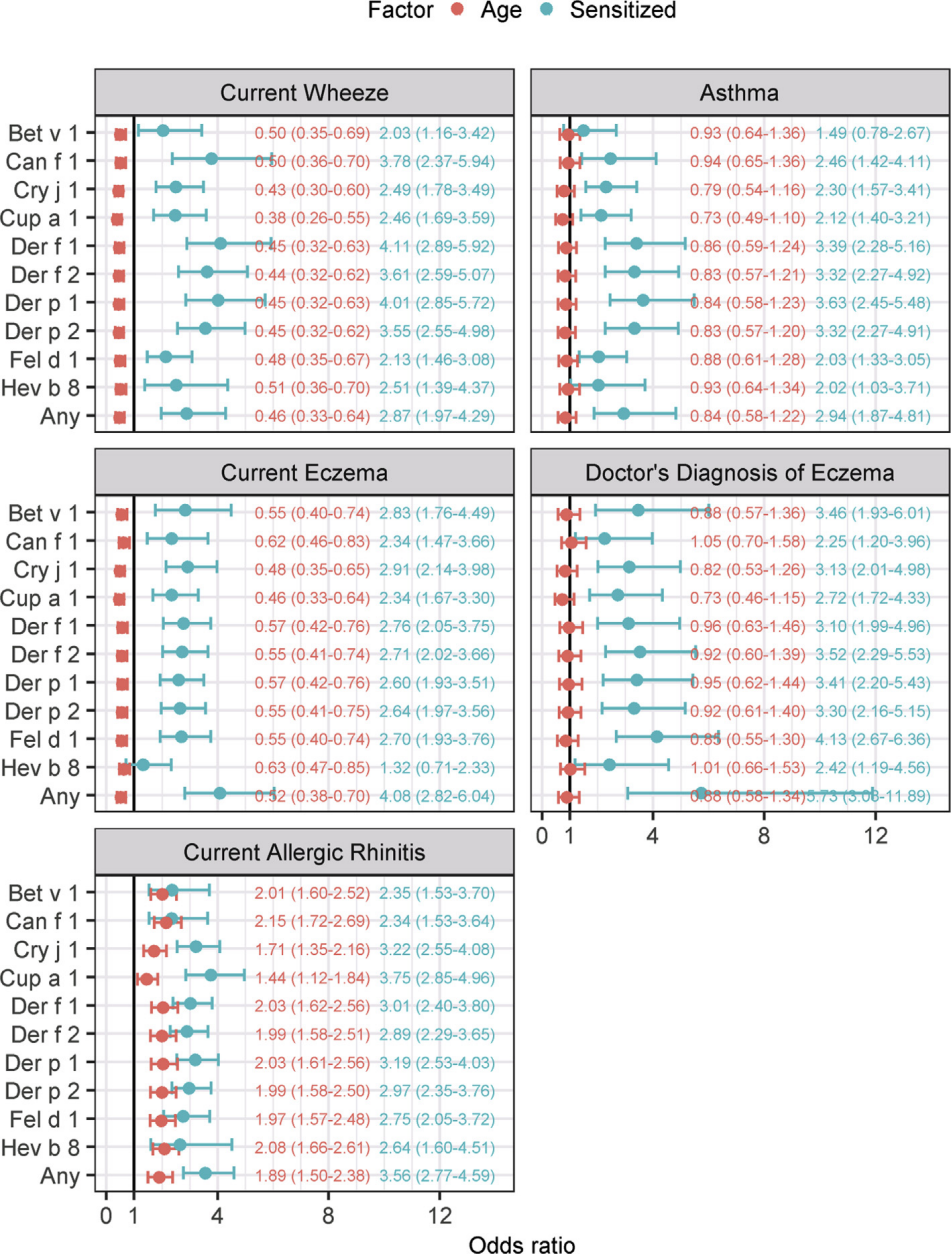
Odds ratios in logistic regression with 95% confidence intervals. An odds ratio greater than 1 indicates that the diagnosis is more common among sensitized subjects or subjects aged 9 years. A confidence interval not containing 1 indicates that the explanatory variable has a statistically significant association with the diagnosis. The bold line on the x-axis indicates an odds ratio of 1. House dust mite (HDM) sensitization (Der f/p) was more prevalent than pollen sensitization in subjects with current wheeze and asthma. Cup a 1 had the highest odds ratio for current allergic rhinitis. In terms of age, the odds ratio was well below 1 for current wheeze and current eczema, indicating that these diseases tend to lessen with age

**Fig. 6:**
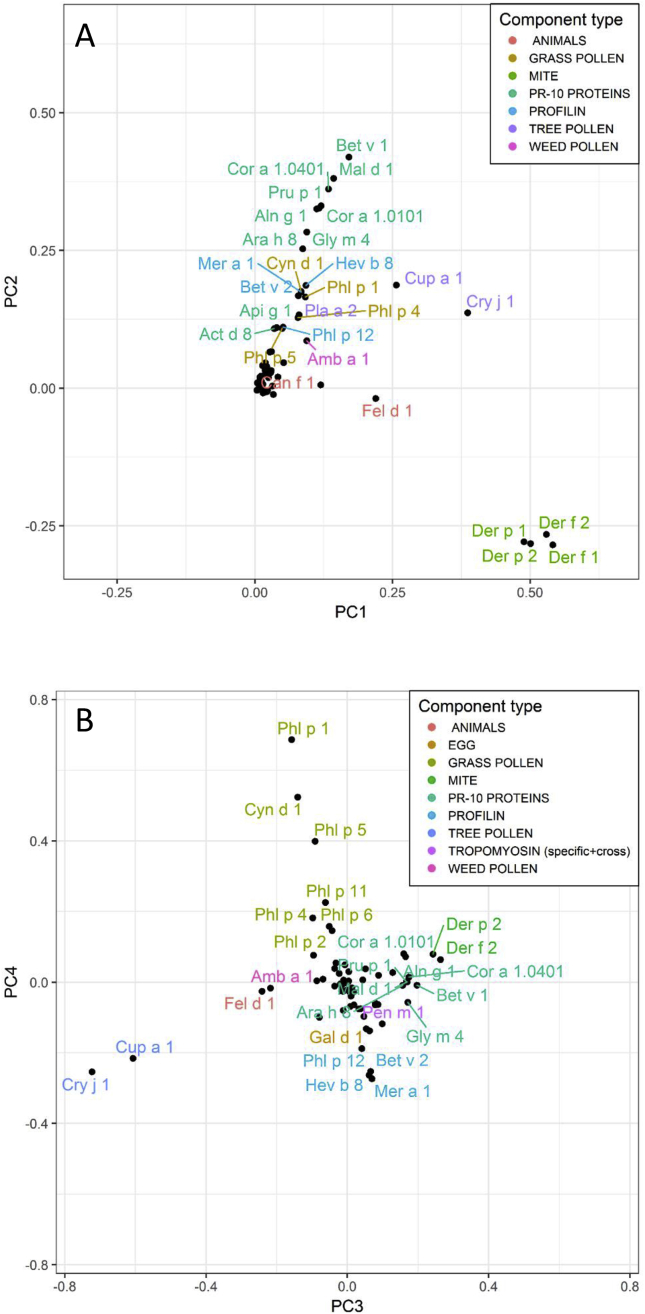
Loading plots from principal component analysis. (A) PC 1 vs PC 2 showed major allergen component correlation patterns in all children based on ImmunoCAP ISAC results at ages 5 and 9 years. (B) PC 3 vs PC 4 showing secondary allergen component correlation patterns. PC 3 and 4 are perpendicular to PC 1 and 2 and are the latent variables explaining the third and fourth largest variations in the dataset, respectively. Note the clustering of related components, such as the mite components, to which cosensitization is common. Coloring is used to highlight the correlation between the related components

McNemar's test for paired data was used to identify components to which sensitization increased with age. A Bonferroni-corrected p-value of 0.05/90 = 0.00056 was considered statistically significant.

Logistic regression analysis was used to assess the importance of age and sensitization to selected components as risk factors for allergic disease (the disease state was the dependent variable, and sensitization and age were explanatory variables). Age was encoded nominally as either 5 years or 9 years. "Any" sensitization referred to sensitization to one or more of the components (Bet v 1, Can f 1, Cry j 1 Cup a 1, Der f 1, Der f 2, Der p 1, Der p 2, Fel d 1 or Hev b 8, which are considered to be important allergen components from a clinical perspective in Japan).

The data were analyzed using principal component analysis (PCA)^18^ to identify any correlation between the allergen components and commonalities in allergic sensitization development between the subjects in the two age groups. PCA is a latent variable projection onto a lower-dimensional orthogonal subspace. The loadings of the first principal component describe the direction in the original space maximizing the explained variance, and the loadings of the second principal component describe the direction of maximal variance or the residuals after removing the variation along the first component. The main results of PCA are commonly presented graphically as scores, the coordinates of a sample in the new coordinate system, loadings, and the relative contributions from the original variables to the new coordinate axes. A biplot combines the scores and loadings to reveal the correlation structure of multidimensional data sets. In the score plots, each child contributes two markers, one for age 5 years and the other for age 9 years. The distribution of markers at a given time point, together with the loading values for the corresponding principal components, describe the sensitization patterns at that time point. A change in the marker positions between the time points describes the development of sensitization patterns over time. For a thorough introduction to PCA, see Jackson (1991). Before the PCA, the data were log-transformed using the formula, $z_{ij}=\ln\left(1+x_{ij}\right)$, where $x_{ij}$ refers to the measured value of subject *i* and allergen component *j*. Only allergen components with at least three positive results (>0.3 ISU) were included in the analysis, resulting in 66 allergen components being selected. Log-transforming the data reduces the impact of a small number of very strong results by normalizing the heavy-tailed distribution. R version 3.5.0 (2018-04-23) was used for data processing and analysis.

## Results

### Study population and prevalence of allergic features

Of the 1550 neonates enrolled in the T-Child study, 984 and 729 subjects aged 5 years and 9 years, respectively, were surveyed with a questionnaire and submitted a blood sample for analysis (Fig. 1). The baseline characteristics are described in Table 1. Maternal allergic history was found in around 60% of subjects and about 50% had a family income >8,000,000 yen. ISAC results were available for 651 subjects for both time points. The prevalence of current allergic rhinitis increased considerably from 10.6% at age 5 years to 31.2% at age 9 years (see Fig. 2). On the other hand, the prevalence of current asthma was relatively stable at the two time points (9.7%–9.4%) while the prevalence of current wheeze decreased (17.0%–9.9%). Furthermore, the prevalence of sIgE sensitization also increased from 57.8% at age 5 years to 74.8% at age 9 years. On the other hand, the prevalence of current eczema and diagnosis of eczema decreased from 21.1% to 30.3% at age 5 years to 15.9%, and 26.7% at age 9 years, respectively.

**Table 1:** Baseline characteristics.

	n	N^a^	%
Age 5 years dataset (N = 984)			
Female sex	494	984	50.2
Birth weight <2500 g	120	982	12.2
Gestational age <37 week	89	984	9
Maternal age ≥ 35 years	422	983	42.9
Cesarean delivery	251	982	25.6
Maternal education level, university	447	943	47.4
Income, >8,000,000 yen	429	897	47.8
Maternal allergy history	563	940	59.9
Age 9 years dataset (N = 729)			
Female sex	365	729	50.1
Birth weight <2500 g	82	728	11.3
Gestational age <37 week	59	729	8.1
Maternal age ≥ 35 years	317	728	43.5
Cesarean delivery	178	727	24.5
Maternal education level, university	337	697	48.3
Income, >8,000,000 yen	316	664	47.6
Maternal allergy history	427	701	60.9

N: Number of participants.

N^a^: Number of participants without missing values

### sIgE sensitization patterns to ISAC components at both time points

Fig. 3 shows the frequency of sIgE sensitization to the allergen components. Among food allergens, Gal d 1 sensitization was found in 0.8 subjects aged 9 years. Jug r 1, 2, and 3-sensitization was found in 1.2%, 8.0%, and 0.4%, respectively, of the subjects at this age. IgE sensitization to Ara h 2 (peanut) and Tri a 19.0101 (ω-5 gliadin, wheat) was found in 2.2% and 0% of the subjects, respectively, at age 9 years. Bal g 7 (cockroach; a type of tropomyosin) sensitization was found in 1.1% of subjects at age 5 years and 2.1% of subjects at age 9 years. Among the PR-10 protein-related allergens, the proportion of IgE sensitization to Bet v 1 (birch) was 2.2% at age 5 years and 13.9% at age 9 years. In subjects aged 9 years, increased sensitization to Mal d 1 (apple), Pru p 1 (peach), and Gly m 4 (soy) was also evident. With regard to airborne allergens, the subjects were also highly sensitized to Cry j 1 (Japanese cedar) and Cup a 1 (Arizona cypress) (32.8% and 12.3% at age 5 years and 57.8% and 46.1% at age 9 years, respectively). The prevalence of IgE sensitization to Der f 1 (mite) at ages 5 and 9 years was 42.1% and 54.3%, respectively. Fel d 1 and Can f 1 sensitization was found in 26.2% and 9.2% subjects at age 9 years, respectively. Sensitization to Mus m 1, another animal allergen, was found in 0.8% of the subjects at age 5 years and 1.0% of the children at age 9 years. Table 2 lists the components showing a statistically significant increase in sensitization from age 5 year to 9 years according to McNemar's test. Among the subjects already sensitized at age 5 years, an increase was more common than a decrease in the measured sensitization value at age 9 years.

**Table 2:** Results from McNemar's test on differences in sensitization between ages 5 and 9 years.

Component	N5 (−)9 (−)	N5 (+)9 (−)	N5 (−)9 (+)	N5 (+)9 (+)	*P*-value
Bet v 1	560	1	76	14	<0.0001
Aln g 1	591	0	55	5	<0.0001
Cor a 1.0101	585	0	60	6	<0.0001
Cor a 1.0401	573	1	66	11	<0.0001
Mal d 1	571	1	67	12	<0.0001
Pru p 1	588	1	50	12	<0.0001
Gly m 4	607	0	36	8	<0.0001
Ara h 8	605	0	39	7	<0.0001
Api g 1	633	0	16	2	0.0002
Cyn d 1	567	1	70	13	<0.0001
Phl p 1	583	2	50	16	<0.0001
Phl p 4	582	3	49	17	<0.0001
Phl p 5	608	4	36	3	<0.0001
Cry j 1	271	5	171	204	<0.0001
Cup a 1	350	1	227	73	<0.0001
Pla a 2	578	4	52	17	<0.0001
Amb a 1	570	4	50	27	<0.0001
Fel d 1	477	6	92	76	<0.0001
Can f 1	586	2	26	37	<0.0001
Alt a 1	625	0	19	7	<0.0001
Der f 1	300	4	80	267	<0.0001
Der f 2	342	3	91	215	<0.0001
Der p 1	319	6	83	243	<0.0001
Der p 2	345	5	93	208	<0.0001
Bet v 2	607	3	29	12	<0.0001
Hev b 8	589	3	45	14	<0.0001
Mer a 1	599	4	34	14	<0.0001
Phl p 12	625	1	18	7	0.0002

N5(−)9(−) is the number children not sensitized at any time point.

N5(+)9(−) is the number of children sensitized at age 5 years but not sensitizedat 9 years. N5(−)9(+) is the number of children sensitized only at age 9 years, N5(+)9(+) is the number of children sensitized at both time points.

Among the subjects already sensitized at age 5 years, an increase in the measured sensitization value at age 9 years was more common than a decrease

### sIgE sensitization patters in relation to allergic features

The prevalence of current wheeze, eczema, and the diagnosis of eczema, asthma, and rhinitis were higher in the sensitized, than in the non-sensitized, subjects (Fig. 4). Among the latter, the prevalence of current wheeze, eczema, and asthma decreased between the two age groups. Among sensitized children, wheeze and current eczema also decreased, but less markedly. The prevalence of the diagnosis of eczema and asthma remained largely unchanged in the sensitized group. However, Bet v 1-sensitized subjects showed a higher prevalence of current eczema than the Cri j 1- sensitized subjects. Rhinitis increased with age in both groups although the increase was greater in the sensitized group. Approximately 75% of subjects at age 9 years who had been sensitized to Cry j 1 or Der f 1 had rhinitis. The odds ratio was between 2 and 4 for all allergic diseases in logistic regression, corroborating these observations (Fig. 5). House dust mite (HDM)-sensitization (Der f/p) was more prevalent than pollen-sensitization in subjects with current wheeze and asthma. For current eczema and the physician's diagnosis of eczema, sensitization to any of the selected components was associated with the highest odds ratio, indicating a strong association with sensitization in general and a weak association with any particular allergen component. Cup a 1 had the highest odds ratio for current allergic rhinitis.

In terms of age, the odds ratio was well below 1 for current wheeze and current eczema, indicating that these diseases tend to lessen with age.

### Relationship of allergen components in principal component analysis

**Fig. 7:**
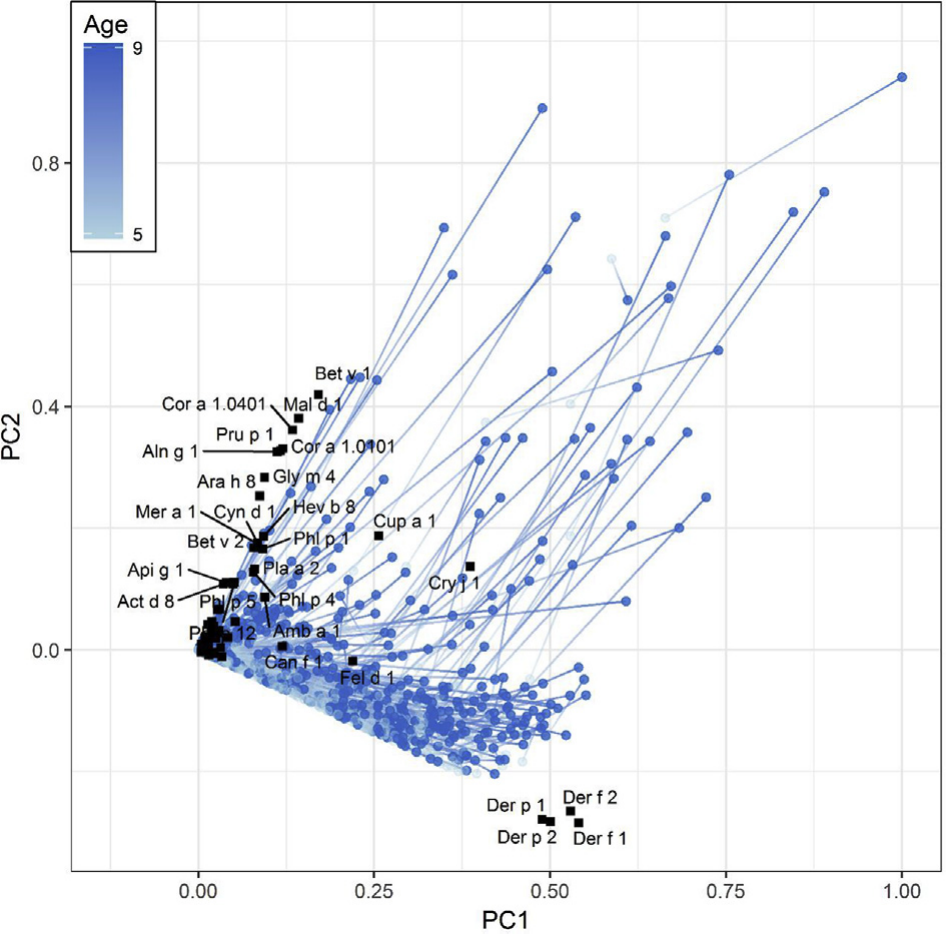
Sensitization development in 651 children. Black markers represent loading values, light-blue and blue markers connected with a gradient line represent the sensitization of an individual subject at ages 5 and 9 years, respectively. There was a general trend in sensitization development, first, mostly towards the mite component, and thereafter towards the PR10 components and tree pollen

The results of the principal component analysis are summarized in Fig. 6, Fig. 7. PCA loading plots revealed the major sensitization patterns (Fig. 6A) as well as more subtle sensitization patterns (Fig. 6B), confirming the association between the allergen components. Components that appeared close together on a loading plot were highly correlated. Mite components clustered around the principal component (PC) 1 (slightly negative on PC2), indicating that the subjects were often multiply sensitized or not sensitized at all to mite components. PR-10 cross-reactive components clustered around PC 2 almost orthogonally to the mite components, indicating that sensitization to the PR-10 cross-reactive components was independent of sensitization to the mite components, i.e., there was no information about whether a subject sensitized to mite components might also be sensitized to PR-10 cross-reactive components (see Fig. 6A). The components, Cry j 1, Cup a 1, Fel d 1, and Can f 1 appeared in the region between PR-10 and the mite components, indicating that subjects sensitized to any of these also often had cross-reactivity to either PR-10 or mite components or both. PC 3 and PC 4 were dominated by Cry j 1, Cup a 1, and the grass pollen components (Fig. 6B). Again, sensitization to grass components seemed to be unrelated to sensitization to cedar or cypress. The food components, to which sensitization was rarer, clustered close to the origin with no easily discernible structure. A biplot with scores (each marker corresponding to a subject at a certain time point, with the gradient line connecting the measurements at ages 5 and 9 years for each subject) and loadings (Fig. 7) showed the subjects becoming increasingly sensitized with age, as reflected in the tendency of the subjects to move away from the origin with age. Sensitization was roughly proportional to the distance from the origin of the coordinates, either by virtue of multiple sensitizations or a high degree of sensitization. Some subjects developed sensitization, first to mite components (the first marker between the origin and mite cluster) and later to pollen (the second marker above the mite cluster parallel to the pollen cluster) while others developed sensitization to pollen only or mite and pollen. However, the subjects rarely developed sensitization to pollen before developing sensitization to mite.

## Discussion

To the best of our knowledge, the present general, prospective, longitudinal study may be the first epidemiological analysis of changes in IgE sensitization to multiple allergen components before and after elementary school enrollment among Japanese children. We found that the prevalence of current rhinitis increased considerably in children from age 5 years–9 years. Furthermore, IgE sensitization to Japanese cedar pollen and HDM was common in the children at age 5 years and increased over time. In addition, sensitization was clearly associated with allergic disease. The clustering pattern seen in mite component sensitization was independent of the sensitization pattern to PR-10 cross-reactive components. Some subjects developed sensitization to mite components first and to pollen components later while others developed sensitization to pollen alone or mite and pollen together. Interestingly, none of the subjects developed sensitization to pollen before sensitization to mite.

Among European studies of IgE sensitization patterns among children, the Manchester Asthma and Allergy Study (MAAS) study reported that the prevalence of positive IgE sensitization to any components by ImmunoCAP ISAC among subjects aged 11 years was 51.6%.^19^ In the Netherlands, 48.9% of children showed IgE sensitization to some type of allergen in 2014.^20^ The German Multicentre Allergy study (MAS) found that IgE sensitization to Phl p 1 and Bet v 1 was the most prevalent (≥20%), followed by IgE sensitization to Fel d 1, Phl p 5, Der p 2, Der p 1, Pla a 2 (10%–20%), Phl p 2, Alt a 1, Phl p 11, and Act d 2 (<10%) among subjects aged 10 years.^21^ The BAMSE cohort study in Sweden showed the prevalence of IgE sensitization to cat was 6.8%, 13.9%, and 19.8% and that to dog was 5.1%, 11.6%, and 22.9%, in subjects aged 4, 8, and 16 years, respectively.^22^ In Italy, IgE sensitization to Der f 1 and Der p 1 developed in more than 50% of school-aged children,^24^ and IgE sensitization to HDM was considered to be common in the general population. Posa et al. found that early onset of sensitization to mite was associated with later polysensitization based on data obtained from subjects aged 0–20 years.^26^ Howard et al. identified several clusters of allergy components using ImmunoCAP ISAC, showing that only HDM formed a cluster at an early age and remained unchanged to age 16 years.^27^ This finding was similar to ours, which showed that sensitization to mite components was independent of sensitization to PR-10 cross-reactive components, and that some subjects developed sensitization to mite components first, as seen in our PCA analysis results. It should be borne in mind that mite is an indoor allergen, whereas pollen is an outdoor allergen. Exposure pathways might therefore differ. Bet v 1-sensitized subjects showed a higher prevalence of eczema than Cri j 1- sensitized subjects, possibly due to the stronger tendency of Bet v 1-sensitization to occur via skin than Cri j 1-sensitization. Sensitization to PR-10-related allergen components, Mal d 1, Pru p 1, and Gly m 4, increased in children at age 9 years, indicating an increased prevalence of pollen-food allergy syndrome (PFAS), in line with the findings of a previous study demonstrating an increase in the prevalence of fruit allergy from ages 4 years–6 years in Japan.^28^

Our study populations showed a very low IgE response to cockroach, in contrast to children in the US. Do et al. reported that the prevalence of cockroach allergy ranged from 17% to 41% in the US, where cockroach allergens were found in 85% of inner-city US homes, and 60%–80% of inner-city children with asthma showed sensitization to cockroach.^23^

Tham et al. noted that IgE sensitization to HDM was extremely high in Singapore (70% to >90%) and Taiwan (85%–90%) among children with an allergic disease,^9^ and we found that IgE sensitization to HDM was approximately 50% among Japanese children. Kitazawa et al. reported that Der f was found in bed sheets and on the floor in all their participants’ homes in Japan.^25^ The Japanese population are highly exposed to HDM in daily life, and consequently show a high proportion of IgE sensitization to HDM.

In 2003, Okuda et al. reported the prevalence of Japanese cedar pollinosis to be 12.8% in children aged 3–9 years and 20.2% in children aged 10–19 years.^29^ The prevalence of Japanese cedar pollinosis appears to have increased over the decades. The 9-year-old subjects in this study were highly sensitized to any allergen components (74.8%), and pregnant women in a large scale birth cohort study in Japan (JECS) also showed high sensitization to any allergens (73.9%).^7^ The almost equal prevalence of allergen sensitization among young adults and 9-year-old subjects in Japan suggests that the adult sensitization patterns might have started from elementary-school age. An Italian cross-sectional study demonstrated that the prevalence of IgE sensitization varied among age groups.^24^ The MAS Study described the development of IgE sensitization to mite allergen from birth to 20 years and demonstrated that the prevalence of mite sensitization increased with age.^26^ The long-term trajectory of IgE sensitization in Japan awaits further clarification in future studies.

Cross-reactive carbohydrate determinants (CCD) are protein-linked carbohydrate structures underlying serum cross-reactivity to a variety of allergens.^30^ Some of our subjects might have been falsely positive to Cyn d 1, Cry j 1, Jug r 2, Pla a 2, Cup a 1, and Pul p 3 due to CCD. In fact, the Jug r 2 sensitization rate was 8.0% in subjects aged 9 years. However, Jug r 2 is thought to have a low prevalence of clinical reactivity.^31^

Interestingly, the prevalence of current asthma was relatively stable after five years, and current wheeze decreased between ages 5 and 9 years in our study. The 2017 Japanese asthma guidelines also indicate a decrease in asthma prevalence among Japanese children.^32^ Most subjects with preschool-age asthma onset achieved good control of their symptoms or experienced a decreased in severity over five years partially due to the improved treatment provided by the guidelines and the higher efficacy of anti-inflammatory drugs.^33^ although some of the subjects may have had early onset transient asthma. One of the limitations of the present study was that the details of their treatment were unable to be obtained by the questionnaire used.

Our study has a number of limitations. First, the fixed ImmunoCAP ISAC panel used with the subjects at age 9 years was updated from the version used with the subjects at age 5 years. The two different ISAC kits were tested for intrakit validity, and both panels contained ninety components. The EAACI has published a molecular allergology user's guide.^4^ D'Urbano et al. reported that the ImmunoCAP ISAC is able to predict milk and egg allergy well in children with positive food challenge test results.^34^, ^35^ Heaps et al. reported that the ISAC array was useful for diagnosing previously unrecognized sensitizations causing idiopathic anaphylaxis.^36^ ImmunoCAP ISAC and whole-allergen CAP equally diagnose grass and cypress pollen allergy.^37^ Multiplex allergen component testing yielded additional information on about 60% of patients in Italy.^38^ ImmunoCAP ISAC was used to measure the outcome of a clinical trial of house dust mite sublingual immunotherapy.^39^ A recent review stated that ImmunoCAP ISAC was an useful tool in the clinical setting^40^ and that the evaluation of IgE sensitization by ImmunoCAP ISAC was suitable not only for epidemiological studies, but also for clinical studies. Second, the subjects in the present study were all residents of the Tokyo metropolitan area; we were therefore unable to examine the differences between children in urban versus rural areas. An Austrian study found that the sensitization prevalence differed among populations in urban, rural, and alpine regions.^41^ In Japan, a large-scale, general, birth cohort study, called the JECS,^42^, ^43^ is following about 100,000 children nationwide and collecting blood samples from the parents and their children to confirm IgE sensitization patterns in various areas. Following up this T-CHILD Study will also reveal the trajectory of IgE sensitization patterns to multiple components in Japan and enable us to compare the findings with those of studies done in the other countries.^27^, ^44^, ^45^

## Conclusion

The prevalence of asthma and atopic dermatitis reached a plateau at age 5 years while that of rhinitis increased drastically from age 5 years–9 years. Sensitization patterns to allergen components differed by age and type of allergic disease. Sensitization to PR-10 components in particular increased dramatically by age 9 years.

## Declarations

### Ethics approval

The study was approved by the Institutional Review Board of the NCCHD in Tokyo, Japan (IRB number: 334, 341, 472, and 614). Informed consent was obtained from all participants.

### Consent to publish

Not applicable.

### Potential conflicts of interest related to this study

KYH received travel expenses from Thermo Fisher Scientific. MB and MÅ are employees of Thermo Fisher Scientific (Phadia AB). The other authors have no conflicts of interest to declare.

### Availability of data and materials

Not applicable.

### Authorship contribution

KYH, TF, and MN did the data sampling. KYH, YO and MB contributed to the study design. MA and LY did the statistical analysis. KYH wrote the first draft of this manuscript. All the authors contributed to, and agreed with final version of, the manuscript.

### Financial support

This study was supported by a grant from the National Center for Child Health and Development (26-18).
